# Evaluation of PIMA^TM^ CD4 System for Decentralization of Immunological Monitoring of HIV-Infected Patients in Senegal

**DOI:** 10.1371/journal.pone.0154000

**Published:** 2016-05-11

**Authors:** Babacar Faye, Moustapha Mbow, Mame Cheikh Seck, Babacar Mbengue, Djiril Wade, Makhtar Camara, Cathy Cissé, Salimata Guèye Diouf, Babacar Ndao, Audrey Djibo, Maguette Dème Sylla Niang, Tandakha Ndiaye, Michael P. Grillo, Alioune Dièye

**Affiliations:** 1 Military Hospital of Ouakam, Dakar, Senegal; 2 Immunology Department, Faculty of Medicine, Pharmacy and Odontology of the Cheikh Anta Diop University of Dakar, Senegal; 3 Senegalese AIDS Army Program, Dakar, Senegal; 4 Immunology Department of the Laboratory of Bacteriology and Virology of Aristide le Dantec University Hospital of Dakar, Senegal; 5 Parasitology Department, Faculty of Medicine, Pharmacy and Odontology of the Cheikh Anta Diop University of Dakar, Senegal; 6 South Garrison Medical Centre, Dakar, Senegal; 7 Department of Defense HIV/AIDS Prevention Program, Naval Health Research Center, San Diego, CA, United States of America; Rush University, UNITED STATES

## Abstract

**Background:**

HIV infection is a concern in the army troupes because of the risk behaviour of the military population. In order to allow regular access to CD4^+^ T cell enumeration of military personnel as well as their dependents and civilians living with HIV, the Senegalese Army AIDS program is implementing PIMA^TM^ Alere technology in urban and semi-urban military medical centres. Validation such device is therefore required prior their wide implementation. The purpose of this study was to compare CD4^+^ T cell count measurements between the PIMA^TM^ Alere to the BD FACSCount^TM^.

**Methodology:**

We selected a total of 200 subjects including 50 patients with CD4^+^ T-cells below 200/mm^3^, 50 between 200 and 350/mm^3^, 50 between 351 and 500/mm^3^, and 50 above 500/mm^3^. CD4^+^ T-cell count was performed on venous blood using the BD FASCount^TM^ as reference method and the PIMA^TM^ Point of Care technology. The mean biases and limits of agreement between the PIMA^TM^ Alere and BD FACSCount^TM^ were assessed with the Bland-Altman analysis, the linear regression performed using the Passing-Bablok regression analysis, and the percent similarity calculated using the Scott method.

**Results:**

Our data have shown a mean difference of 22.3 cells/mm^3^ [95%CI:9.1–35.5] between the BD FACSCount^TM^ and PIMA^TM^ Alere CD4 measurements. However, the mean differences of the two methods was not significantly different to zero when CD4^+^ T-cell count was below 350/mm^3^ (P = 0.76). The Passing-Bablok regression in categorized CD4 counts has also showed concordance correlation coefficient of 0.89 for CD4^+^ T cell counts below 350/mm^3^ whilst it was 0.5 when CD4 was above 350/mm^3^.

**Conclusion:**

Overall, our data have shown that for low CD4 counts, the results from the PIMA^TM^ Alere provided accurate CD4^+^ T cell counts with a good agreement compared to the FACSCount^TM^.

## Introduction

HIV infection is a concern for the Senegalese army forces because of the high mobility of soldiers frequently engaged in external peace keeping missions, the task of risk of the military population, and the high prevalence of HIV in Casamance post conflict area [1] where there is a high concentration of the military population. The fight against AIDS in the Army as well as in the general population is in line with the safety of the troops. In an effort to support people living with HIV (PLHIV), efforts have been made to better access the clinical outcomes and ensure accurate diagnostics.

CD4^+^ T-cell enumeration is frequently used to assess the immunity level of HIV-positive patients, determining their eligibility for antiretroviral therapy (ART) [2, 3]. In most clinical settings, CD4^+^ T-cell enumeration is performed using flow cytometry-based systems, such as FACSCount^TM^ (Becton Dickinson, San Jose, CA), and is considered the reference method because of its precision, accuracy, and reproducibility [4–8]. However, costly maintenance, the need for well-trained laboratory staff and a cold chain to ship and store reagents can limit its use. Based on these field challenges, the AIDS program of the Senegalese Army Forces, in collaboration with Senegalese National AIDS Program and United States Department of Defense HIV/AIDS Prevention Program (DHAPP), is implementing the PIMA^TM^ Point-of-care (POC) CD4^+^ T-cell enumeration technology (Alere, Jena, Germany) in urban and semi-urban military bases and medical facilities where military personnel, their dependents and civilians living with HIV are being cared for and treated at these sites. Adoption of such non-flow cytometry-based CD4^+^ T-cell counting device will allow regular access to CD4^+^ T-cell enumeration in rural and semi-urban sites in Senegal. Utilizing the PIMA^TM^ Point-of-care (POC) CD4^+^ T-cell enumeration technology will also supports the National and Army AIDS programs strategies to control HIV infection and its transmission. However, since laboratory and field evaluation of the CD4 systems is part of the prequalification of a diagnostic program, validation of POC technologies is therefore required prior their wide implementation [9].

The purpose in this study was to compare T-cell count measurements between the PIMA^TM^ CD4 (Alere, Jena, Germany) to the BD FACSCount^TM^ which has been extensively validated in resource-limited settings [10–11].

## Material and Methods

### Study population and sites

Study participants consisted of adults HIV-infected patients who were being followed up through the Senegalese National AIDS Program and Army AIDS Program from July 2014 to May 2015. Whole blood samples were collected in K3 EDTA-containing tubes from 200 individuals including 183 HIV-infected patients and 17 HIV-negative volunteers who agreed to participate in the study. These facilities include the military bases of Tambacounda, Ziguinchor, Kolda as well as the Military Hospital of Ouakam (MHO) which is the main military health facility in Senegal located west of the Dakar. Subjects were selected based on their CD4^+^ T-cell count as follows: 50 patients below 200/mm^3^ 50 patients between 200 and 350/ mm^3^, 50 patients between 350 and 500/ mm^3^, and 50 patients above 500/ mm^3^.

CD4^+^ T-cell enumerations were performed at the medical biology laboratory of the MHO that hosts the laboratory platform of the Senegalese Military AIDS Program.

### Ethics statement

This study was approved by the National Ethical Committee of the Ministry of Health of Senegal. The ethics committee waived the need for written informed consent from the study participants since we used excess of blood samples from anonymous participants who underwent their routine CD4^+^ T-cell count.

### CD4^+^ T-cell enumeration

CD4^+^ T-cell counts were assessed in whole blood samples within 4 hours of blood collection using the FASCount^TM^ (Becton Dikinson) analyzer and the PIMA^TM^ Point of Care technology.

The BD FACSCount^TM^, equipped with a single platform possesses a green laser combined with built-in software uses two-color monoclonal antibodies (mAb) reagents in twin tubes containing calibrated beads, with additional control beads. Briefly, 50μl of EDTA whole blood was added to the BD FACSCount^TM^ reagent tubes containing anti-CD3-PE, and anti-CD4-PE-Cy5 or anti-CD8-PE-Cy5. The tubes were capped, vortexed and incubated in the dark at room temperature for 1 hour. After incubation, 50ul of fixative solution was added to reagent tubes, and samples were run on the BD FACSCount^TM^ instrument.

The BD FACSCount^TM^ machine is certified by the Quality Assessment and Standardization of Immunological Measures Relevant to HIV/AIDS (QASI) with the BD FACSCount^TM^ reagents that only provide absolute CD4 counts.

Similarly, the CD4^+^ T-cell measurement with the PIMA^TM^ POC was performed according to manufacturer’s procedure. The samples were run only after the normal and low value control cartridges gave acceptable values. Twenty five microliters of venous blood sample collected in EDTA tube were introduced into the PIMA disposable cartridge containing anti-human CD3-dye1 and CD4-dye2 monoclonal antibodies. The collector was then removed and the cartridge immediately inserted into PIMA analyzer.

### Statistical analysis

Data were entered in Microsoft Access and then exported to SPSS version 17 (IBM), MedCalc 10.0.2.0 (MedCalc Software, Mariakerke), and GraphPad Prism version 5.00 for Windows (GraphPad Software, San Diego California USA) for statistical analysis and graphing. The differences between the two measurements as well as the mean differences of these measurements were computed in SPSS software. One-sample T-test was used to assess the difference between the two measurements by comparing the mean differences to zero. The mean biases and the limits of agreement (LOA) were assessed with the Bland-Altman analysis using the mean differences between the two measurements and the 95% confident limits of the measurement (LOA = mean ± 1.96 SD). The linear regression was performed using the Passing-Bablok regression analysis. Percent similarity between the PIMA^TM^ Alere and BD FACSCount^TM^ was calculated for each sample following the formula: average of methods A and B x 100)/method A (where method A = reference method (BD FACSCount^TM^) and method B = method to evaluate (PIMA^TM^ Alere)) [12].

## Results

### Study population

A total of 200 adults were elected for the present study. The median age was 36 years (range: 19–69) and the female sex represented 69% of the study population. The median (min-max) absolute CD4 counts provided by BD FACSCount and PIMA^TM^ alere were 349 cells/mm^3^ (1–2000) and 332 cells/mm3 (9–2221) respectively.

### Difference between the BD FACSCount^TM^ and PIMA^TM^ Alere CD4 measurements

**Fig 1:**
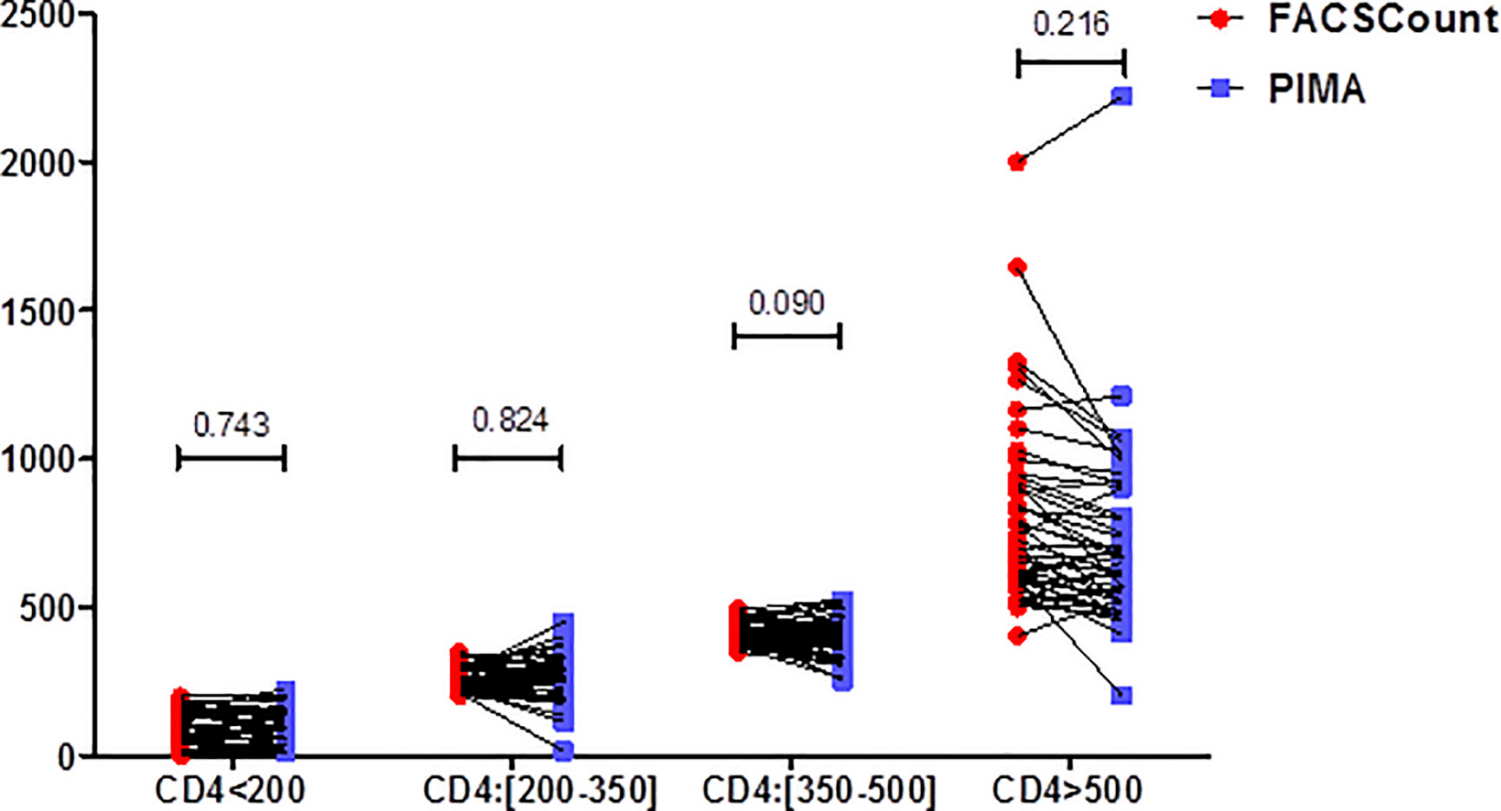
Analysis of the median CD4 counts between the PIMA and the FACSCount. Categories of absolute CD4+ T-cells counts (< 200/mm^3^, [200-350/mm^3^], [351-500/mm^3^], and > 500/mm^3^, in before-after scatter plots comparing the PIMA^TM^ Alere and the BD FACSCount^TM^ are shown. Data are shown as median values. P-values were calculated in SPSS 20 using nonparametric Mann-Whitney U test and the graphing was performed using GraphPad Prism software version 5.00.

The median CD4 counts obtained using PIMA^TM^ Alere analyzer were compared with those obtained from the BD FACSCount^TM^ for the following count categories <200, 200–350, 351–500 and >500 cells/mm^3^. For all CD4 ranges, the differences in median CD4 counts were not significant between the two methods (all p-values > 0.05) (Fig 1).

We also investigated the difference between the two measurements by comparing the mean differences between the two methods to zero using the one-sample T test. Our data have shown that the mean difference of 22.3 cells/mm^3^ [95%CI:9.1–35.5] that was significantly different from zero, indicating that overall, the measurements by the two methods were not similar (P = 0.001) (Table 1). Using the categorized CD4 cell counts, the mean differences were -4,0 [95%CI:-11.9–3.9], -2,7 [95%CI:-20.9–15.4], 19.9 [95%CI:1,6–38,3], and 76,1 [95%CI:32,9–119.4] for CD4 counts < 200/mm^3^, between 200 and 350/mm^3^, between 351 and 500/mm^3^, above 500/mm^3^, respectively. The mean differences of the two measurements to zero was not significantly different for CD4 cell count levels below 200/mm^3^ and between 200 (P = 0.31) and 350/mm^3^ (P = 0.76), indication that there was an agreement for the cell count levels between the two measurements for CD4 counts below 350/mm^3^. Conversely, there was no agreement in the cell count when CD4 counts were between 350 and 500/mm^3^ (P = 0,034) or above 500/m^3^ (P = 0.001) (Table 1).

**Table 1:** Comparison of the mean difference between the CD4 PIMA^TM^ Alere and BD FACSCount^TM^ CD4 measurements.

CD4/mm^3^	Mean Diff	SD Diff	95% confident limits		P-Value
			Upper	Lower	
All	22.3	94.9	35.5	-9.1	0.001
CD4<200	-4.0	27.9	3.90	-12.0	0.311
CD4:[200–350]	-2.8	64.0	15.43	-20.95	0.762
CD4:[350–500]	20.0	64.7	38.35	1.57	0.034
CD4>500	76.2	152.0	119.36	32.96	0.001

Abbreviations: Mean Diff: mean difference between PIMA^TM^ Alere and BD FACSCount^TM^ CD4 measurements; SD Diff: standar deviation difference between PIMA^TM^ Alere and BD FACSCount^TM^ CD4 measurements

**Fig 2:**
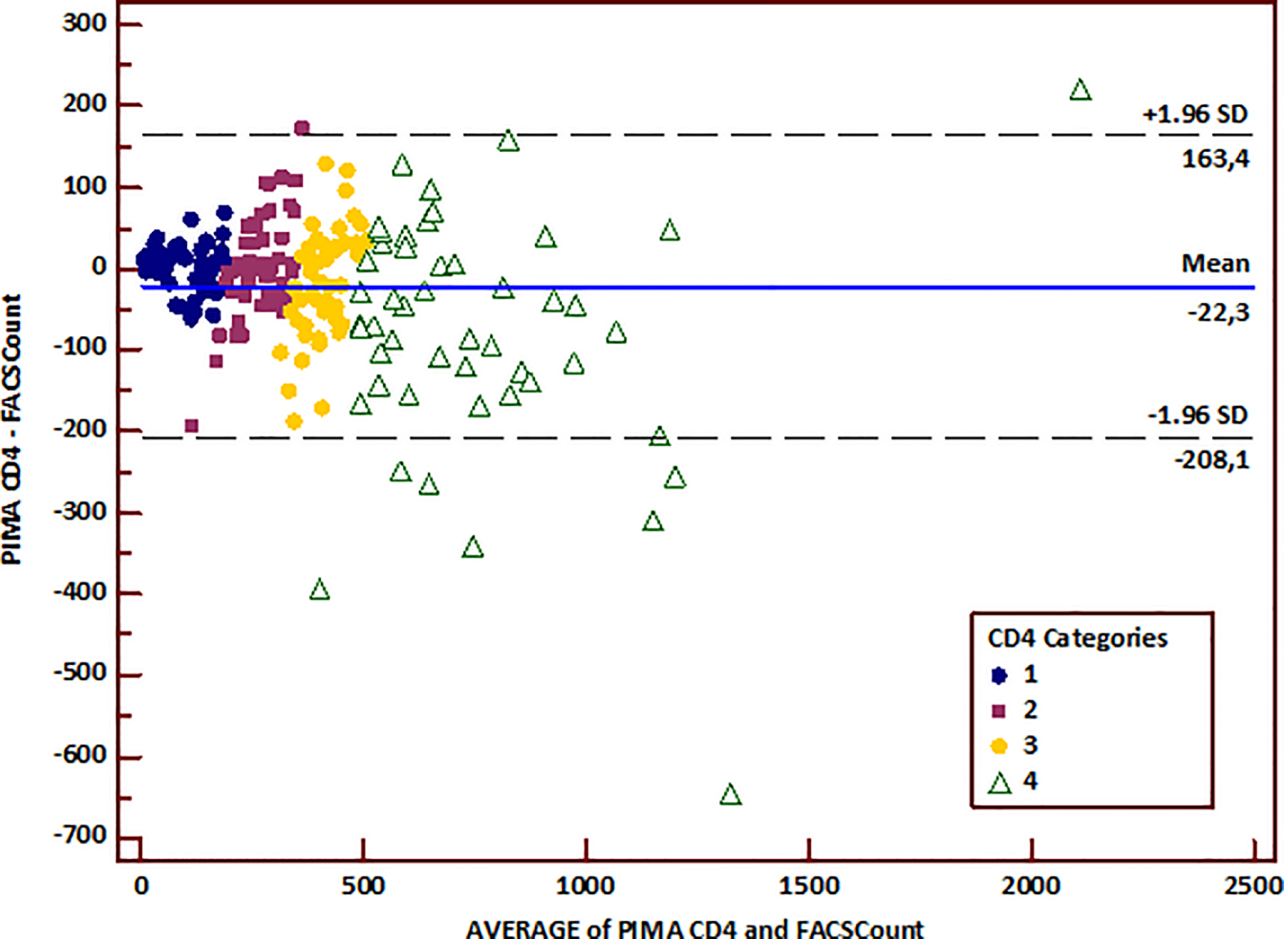
Bland-Altman plot of the whole data. The x-axis represents the average of CD4 count between the PIMA and the BD FACSCount^TM^, and the y-axis represents the bias (difference) between the PIMA^TM^ Alere and the BD FACSCount^TM^. The solid blue line represents the mean of the difference between the two measurements, and the light black lines represent the upper and lower limits of agreement (ULA: mean differences plus and 1,96 x standard deviation of the mean difference; LLA: mean differences minus and 1,96 x standard deviation of the mean difference). Legend for CD4 categories: 1: CD4 T-cells < 200/mm^3^; 2: CD4 T-cells between 200 and 350/mm^3^; 3: CD4 T-cells between 351 and 500/mm^3^; 4: CD4 T-cells above 500/mm^3^.

The difference and mean differences of the counts obtained were plotted using the Bland-Altman method to visualize the bias, i.e. the random fluctuation of measurements around the mean difference line. We found that the average discrepancy between the methods (bias) was higher when the CD4 count was above 350/mm^3^ (Fig 2). In other words, the difference between the two methods became larger as the average increased.

**Fig 3:**
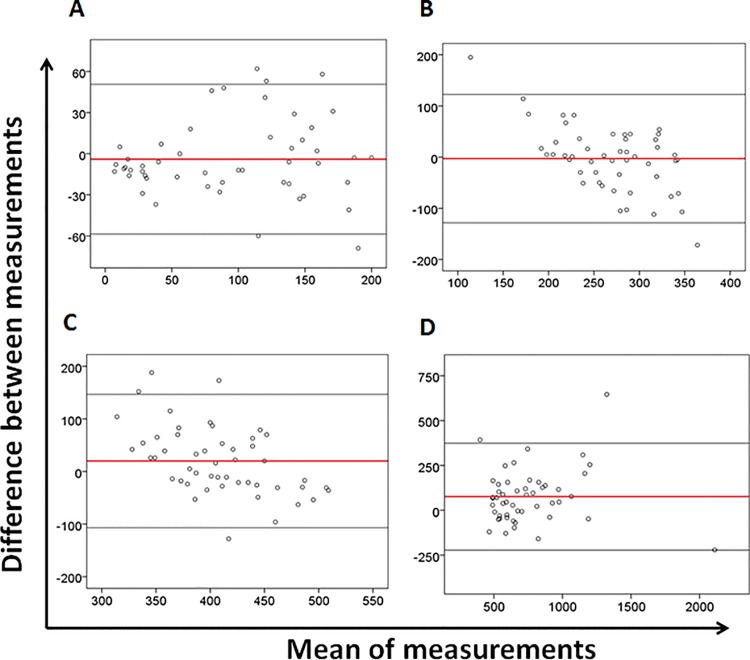
Bland-Altman analysis. Bland-Altman plots of **(A)** CD4 T-cells < 200/mm^3^, **(B)** between 200 and 350/mm^3^, **(C)** between 350 and 500/mm^3^, and **(D)** above 500/mm^3^ according the CD4 cell count levels. For each plot, the x-axis represents the average of CD4 count between the PIMA and the BD FACSCount^TM^, and the y-axis represents the bias (difference) between the PIMA^TM^ Alere and the BD FACSCount^TM^. The solid red line represents the mean of the difference between the two measurements, and the light black lines represent the upper and lower limits of agreement (ULA: mean differences plus and 1,96 x standard deviation of the mean difference; LLA: mean differences minus and 1,96 x standard deviation of the mean difference)

The Bland Altman plot analysis showed an absolute bias of -22 (with lower and higher agreement limits of -208 and 163 respectively) indicating that overall, the PIMA underestimated the BD FACSCount^TM^ by 22 cells/mm^3^. Calculation of the relative bias has shown an inter-essay variation of -0.2%. The Bland-Altman plots also showed that the higher the CD4 T-cell counts were, the wider the limits agreements became (Fig 3; Table 2).

**Table 2:** Comparison of CD4 T-cell counts between Pima CD4 and FACSCount.

	All	CD4<200	CD4:[200–350]	CD4:[350–500]	CD4>500
N	200	50	50	50	50
Intercept [95% CI] (cells/mm3)	11[2–21]	11[3–17]	-203[-429-(-)51]	-410[-881-(-)175]	-14[-168-99]
Slope [95% CI]	0.93[0.89–0.98]	0.97[0.86–1.10]	1.76[1.18–2.61]	1.96[1.41–3.10]	0.91[0.76–1.13]
Concordance ρ_c_ [95% CI]	0.94[0.93–0.96]	0.89[0.82–0.93]	0.48[0.28–0.63]	0.32[0.10–0.51]	0.85[0.75–0.91]
Pearson ρ (precision)	0.95	0.89	0.58	0.39	0.89
Accuracy C_b_	0.99	0.99	0.86	0.83	0.96
Bias [LOA] (cells/mm3)	-22[-208-136]	18[(-)71-108]	2[-123-127]	-17[-149-116]	-80[-376-216]

Abbreviations: N: sample size; 95% CI: 95% of Confidence Interval; LOA: limits of agreement, SD: standard deviation.

No proportional bias was found when the CD4 count was below 200/mm^3^ (P = 0.29). However, there were biases between the two measurements when CD4 counts were above 200mm^3^ (p-value significant for all CD4 levels above 200/mm^3^) (Table 1 and Table 2).

**Fig 4:**
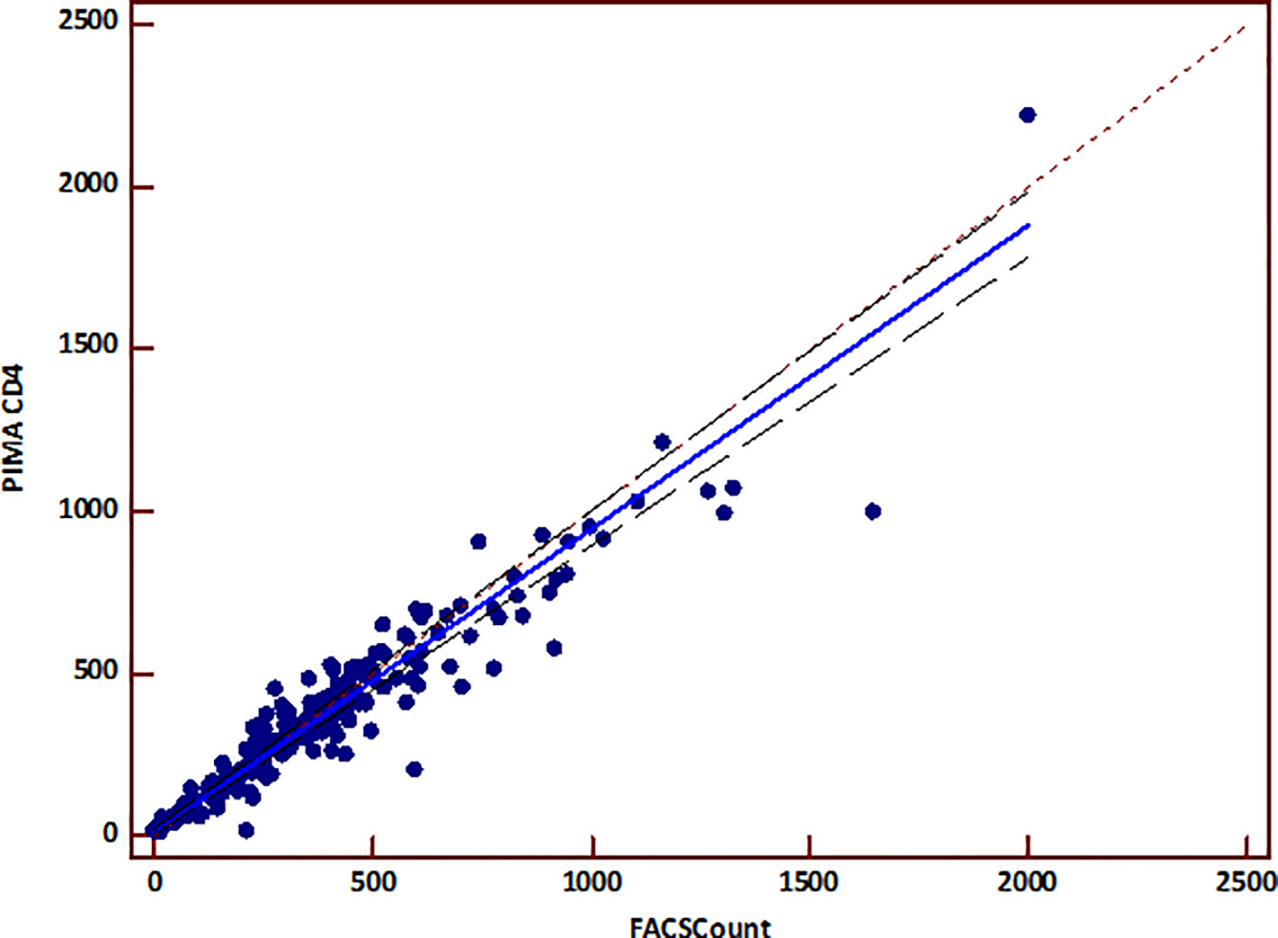
Passing-Bablok regression between PIMA^TM^ Alere CD4 and BD FACSCount^TM^. The x-axis represents CD4 counts provided by the BD FACSCount^TM^ reference and the y-axis represents the CD4 counts provided by the PIMA^TM^ CD4. The solid line represents the regression line, and the dashed line represents the line y = x. The linear equation of the regression is y = 0.9347 x + 11.

The linear regression analysis did not show a proportional bias between the two measurements (Fig 4). The Passing-Bablok regression analysis between the two measurements showed an intercept of 11 cells/mm3 and a slope of 0.93 (Table 2). The corresponding concordance correlation coefficient was 0.94 for PIMA^TM^ Alere, showing a good correlation and concordance between the two methods (Table 2).

The Passing-Bablok regression analysis using categorized CD4 counts also showed concordance correlation coefficient of 0.89 when CD4^+^ T-cell counts were below 350/mm^3^ while it was 0.5 when CD4 was above 350/mm^3^.

## Discussion

As a result of intensive HIV testing campaigns in order for people to know their status, and the well-established importance of getting people living with HIV (PLHIV) in care and offered treatment, Senegal has one of the lowest HIV prevalence in Sub-Saharan Africa. In an effort to improve and decentralize care for PLHIV, there is a needed of better access to CD4^+^ T-cell enumeration. In order to allow regular access to CD4^+^ T-cell enumeration in rural and semi-urban sites in Senegal as well as respond to the National AIDS program and WHO strategy to control HIV infection, the Senegalese Army AIDS program is implementing PMA^TM^ Alere CD4^+^ T-cell enumeration technology in urban and semi-urban military medical centre.

The BD FACSCount^TM^ has been the first instrument offering affordable and cheaper T-cell counts for biological monitoring of HIV-positive patients. However, its high supply and maintenance costs inspired the Senegalese AIDS National Program and the Army AIDS Program to progressively switch toward affordable new POC CD4 counting instrument including the PIMA^TM^ Alere which is able to respond to this demand. Due to the limited precision and accuracy that all instruments might have and that might lead to discordant results, we aimed in this study to validate the PIMA^TM^ instrument using the FACSCount^TM^ as reference instrument. We found that for low CD4 values, the PIMA^TM^ counter provided concordant results compared to the BD FACSCount^TM^.

Our data showed that the PIMA^TM^ analyzer gave comparable median CD4 counts with the reference methodology in different ranges, including below 200, 200–350, 351–500 and above 500 cells/mm^3^. There was no significant difference in the median CD4 counts between the PIMA^TM^ and the FACSCount^TM^. This is consistent with the findings reported by Thakar et *al*. who did not find any significant difference in median CD4 counts obtained by the reference method and the PIMA^TM^ analyzer [13].

However using more conventional comparison method of two quantitative measurements, we assessed the mean difference between the two measurements that appeared significantly different from zero using One-sample T-test. This indicates that there was no perfect agreement between the PMA^TM^ and FACSCount^TM^ measurements. After categorizing CD4^+^ T-cell counts, we found that such difference was not significant for CD4 ranges below 350/mm^3^, highlighting a good agreement between the two measurements for low CD4 counts.

The analysis of the agreement between FACSCount^TM^ and PIMA^TM^ Alere CD4 measurement have shown an absolute bias of -22 cells, indicating that overall, the PIMA^TM^ slightly underestimates the gold standard by 22 cells/mm^3^. This is consist with Myer et *al*. findings in HIV-positive pregnant women showing the PIMA^TM^ POC test to slightly underestimate CD4 cell count relative to flow cytometry based systems by 23 cells/mm^3^, with limits of agreement from -129.2 to 174.6 cells/mm^3^ [14]. As we reported, the majority of studies comparing the Alere PIMA^TM^ analyzer using venous specimens have found that the POC methods underestimate laboratory-based flow cytometry methods [15,16] despite providing still a good correlation with conventional technologies [17]. Nevertheless, the relative bias between the PIMA^TM^ and reference methodology was very low, suggesting a good agreement between the two measurements. Similar low relative bias has also been reported in studies comparing CD4^+^ T-cell counts between the PIMA^TM^ Alere and the BD FACSCount^TM^ [13].

Through the Passing-Bablok regression analysis, we found a good concordance correlation coefficient between the methods for low and medium CD4 values. This is in line with previous findings showing that the PIMA^TM^ CD4 was found to be less precise than the flow cytometry based systems, in particular for CD4 counts below 200 cells/mm^3^ [18]. Previous studies have reported that the PIMA^TM^ device is comparable and interchangeable with the existing CD4 enumeration platforms [13, 15, 19, 20]. In our study, despite the PIMA Counter showing good concordance and correlation with BD FACSCount^TM^, we did only find such evidence in low and medium CD4 counts. This discrepancy might however reflect the significant contribution of experiment conditions.

Consistent with our results, when CD4 counts were above 500/mm^3^, the PIMA^TM^ Alere tended to misclassify patients by 80 cells/mm^3^ (with agreement limits from -376 to 216 cells/mm^3^) toward an underestimation compared with the BD FACSCount^TM^. This suggests that using the PIMA technology, patients who need treatment may not qualify for ART under current guidelines. Nevertheless, the bias was low (< 20 cells/mm3) for CD4 counts below 350/mm^3^.

It has also been reported that the sensitivity and specificity of the PIMA^TM^ Alere was 83.1% and 92.2% respectively when BD FACSCalibur^TM^ was used as gold standard, and 79.4% and 83.4% respectively when BD FACSCount^TM^ was considered as reference method [21]. Moreover, the same study has shown that the PIMA^TM^ and BD FACSCount^TM^ coefficients of repeatability were very different, at 179.2 cells/ml and 67.4 cells/ml respectively, indicating that the tests on the BD FACSCount^TM^ were more repeatable. These results and ours do not fully support that the PIMA^TM^ device is interchangeable with the FACSCount^TM^ CD4 enumeration platform. Since the purpose of point-of-care technologies is to provide wider access to a CD4^+^ T-cell enumeration service in remote rural settings, they may not provide the same sensitivity, specificity and accuracy as the ‘gold standard’ reference tests. Moreover, the principles in the tests themselves, i.e. fluorescence optic imaging versus flow cytometry based system might reflect differences in the precision regarding certain ranges of CD4 counts. Overall, if operators’ contribution on such discrepancies is dismissed, it therefore appears that the PIMA^TM^ technology might need further refinement to improve repeatability. Because of its characteristics and particular interest for use in small health from resource-limited settings, improvement of the PIMA^TM^ Alere is nevertheless a need, especially in light of the new World Health Organizations recommendations on starting treatment as soon as possible [22].

Overall, we have demonstrated that for low CD4^+^ T-cell counts, the results from the PIMA^TM^ Alere obtained from venous blood can provide accurate data which a good agreement with the FACSCount^TM^. Despite PIMA^TM^ Alere CD4 system can be operated easily in resource-limited settings, its wider implementation will require deeper evolution and/or further refinement of its system so that to reach perfect concordance between the PIMA and the existing reference systems.

## Supporting Information

S1 File SPSS Database. (SAV)
